# WIN55,212-2 Attenuates Cognitive Impairments in AlCl_3_ + d-Galactose-Induced Alzheimer’s Disease Rats by Enhancing Neurogenesis and Reversing Oxidative Stress

**DOI:** 10.3390/biomedicines9091270

**Published:** 2021-09-19

**Authors:** Onesimus Mahdi, Samaila Musa Chiroma, Mohamad Taufik Hidayat Baharuldin, Nurul Huda Mohd Nor, Che Norma Mat Taib, Saravanan Jagadeesan, Shamala Devi, Mohamad Aris Mohd Moklas

**Affiliations:** 1 Department of Human Anatomy, Faculty of Medicine and Health Sciences, Universiti Putra Malaysia, Serdang 434001, Malaysia; omahdi2010@gmail.com (O.M.); musasamailachiroma@yahoo.com (S.M.C.); hudamohdnor@upm.edu.my (N.H.M.N.); chenorma@upm.edu.my (C.N.M.T.); mljsaravanan@gmail.com (S.J.); maladevi@upm.edu.my (S.D.); 2 Department of Human Anatomy, College of Medical Sciences, Gombe State University, Gombe 760211, Nigeria; 3 Department of Human Anatomy, Faculty of Basic Medical Sciences, University of Maiduguri, Maiduguri 600230, Nigeria; 4 Faculty of Medicine, Manipal University College Malaysia (MUCM), Melaka 75150, Malaysia

**Keywords:** WIN55,212-2, cognitive functions, aluminium chloride, d-galactose, Alzheimer’s disease, cannabinoid, oxidative stress, neurogenesis

## Abstract

Neurotransmission and cognitive dysfunctions have been linked to old age disorders including Alzheimer’s disease (AD). Aluminium is a known neurotoxic metal, whereas d-galactose (d-gal) has been established as a senescence agent. WIN55,212-2 (WIN), is a potent cannabinoid agonist which partially restores neurogenesis in aged rats. The current study aimed to explore the therapeutic potentials of WIN on Aluminium chloride (AlCl_3_) and d-gal-induced rat models with cognitive dysfunction. Healthy male albino Wistar rats weighing between 200–250 g were injected with d-gal 60 mg/kg intra peritoneally (i.p), while AlCl_3_ (200 mg/kg) was orally administered once daily for 10 consecutive weeks. Subsequently, from weeks 8–11 rats were co-administered with WIN (0.5, 1 and 2 mg/kg/day) and donepezil 1 mg/kg. The cognitive functions of the rats were assessed with a Morris water maze (MWM). Furthermore, oxidative stress biomarkers; malondialdehyde (MDA), superoxide dismutase (SOD), glutathione (GSH) and neurogenesis markers: Nestin and glial fibrillary acidic protein (GFAP) were also evaluated, as well as the histology of the hippocampus. The results revealed that rats exposed to AlCl_3_ and d-gal alone showed cognitive impairments and marked neuronal loss (*p* < 0.05) in their hippocampal conus ammonis 1 (CA1). Additionally, a significant decrease in the expressions of GFAP and Nestin was also observed, including increased levels of MDA and decreased levels of SOD and GSH. However, administration of WIN irrespective of the doses given reversed the cognitive impairments and the associated biochemical derangements. As there were increases in the levels SOD, GSH, Nestin and GFAP (*p* < 0.05), while a significant decrease in the levels of MDA was observed, besides attenuation of the aberrant cytoarchitecture of the rat’s hippocampi. The biochemical profiles of the WIN-treated rats were normal. Thus, these findings offer possible scientific evidence of WIN being an effective candidate in the treatment of AD-related cognitive deficits.

## 1. Introduction

Alzheimer’s disease (AD) is a neurodegenerative disorder commonly present among the elderly presenting with memory and cognitive dysfunction. The key pathological characteristics of AD are the presence of neurofibrillary tangles (NFTs), senile plaques (SPs) and the loss of cholinergic neurons in the basal forebrain in humans [1]. Two main types of AD have been established—early-onset AD which affects people less than 65 years of age [2] while the other type of AD is known as late-onset AD, which affects people older than 65 years [3]. The disease is progressive by nature, whereby the symptoms continue to rise and the quality of life worsens over years. AD develops via three stages; mild, moderate and severe. In its early stages, AD patients suffer mild memory loss, but as the disease progresses, patients lose their ability to carry out daily activities [4]. As of 2010, more than 36 million people were affected by AD worldwide [5] and the number of AD patients globally was predicted to increase to about 106.8 million by 2050. Thus, AD is thought to be a rising and dire public health concern with enormous social and economic impacts among the elderly [6,7].

Aluminium (Al) is ubiquitously distributed in our environment and was found as the third most available metal on earth [8,9,10,11,12]. Al finds its way into nearly every aspect of our daily lives thus, it can be absorbed into the human body easily due to its wide range of uses in many households [11]; it is found in deodorants and medications such as antacids. Once it permeates the human brain, Al alters both fast and slow axonal transports, impedes long-term potentiation, causes inflammatory responses, and induces synaptic and structural anomalies which leads to severe neurodegeneration [13]. Findings have established the link between the neurotoxicity of Al in animals and its contribution to the pathogenesis of neurodegenerative diseases such as AD in humans [14]. Excessive exposure to Al can lead to the overexpression of (amyloid-beta precursor protein) AβPP consequently resulting in the deposition of (amyloid-beta) Aβ plaque on the brain cells, thus making Al a potential alzheimerogenic chemical [15,16]. Al toxicity induces cholinergic terminals to degenerate in the cortex resulting in the reduction of neuronal cells in the brain thus causing learning dysfunctions [17]. d-galactose (d-gal) is a reducing sugar that simply reacts with the free amines of amino acids in peptides and proteins, producing advanced glycation end products (AGEs) [18,19]. Chronic administration of d-gal at low doses has caused changes that mimic natural aging processes in animals, such as oxidative stress [20], cognitive decline [21], decreased immune response [22] and alterations in gene transcription [23]. Neurotoxicity caused as a result of chronic systemic exposure to d-gal in rats has been commonly used as a model for studying the mechanism of AD [24,25]. In addition, experiments involving continuous subcutaneous administration of d-gal in mice have caused a remarkable decrease in acetylcholine (ACh) activities in their brain [26]. The combination of Al and d-gal for mimicking AD-like pathologies and cognitive impairments serves as a good model for studying therapeutics against AD.

WIN55,212-2 (WIN), is a potent synthetic aminoalkylindole cannabinoid that is widely gaining popularity to be used as a tool in cannabinoid research. It was shown to produce a wide range of in vivo effects via the endocannabinoid system observed in Tetrahydrocannabinol (THC) and other cannabinoids [27]. Administration of WIN was shown to partly restore neurogenesis in the hippocampus of aged rats. This synthetic cannabinoid agonist was reported to exert its action through the endocannabinoid system (EC) in the central nervous system. The EC is a neuromodulatory signaling complex that encompasses cannabinoid receptors, their endogenous ligands, and proteins implicated in the formation, transport and degradation of such ligands [28].

In AD, the loss of cholinergic neurons and subsequently, the reduction in cholinergic neurotransmission results in cognitive and behavioural impairments. To date, the only categories of drugs approved by the United States Food and Drug Administration (USFDA) for managing the symptoms of AD are low-affinity N-methyl-d-aspartate (NMDA) antagonist and cholinesterase inhibitors (ChEIs). Memantine which is an NMDA glutamate receptor antagonist acts by decreasing the glutamatergic neuronal excitotoxicity whereas ChEIs, which include rivastigmine, galantamine and donepezil, slow the hydrolysis of acetylcholine released into synaptic clefts, thereby enhancing cholinergic transmission [6,29]. The available treatments can only reduce the AD-associated symptoms [30] or at best slow the progression of the disease by counteracting the neurotransmitter disturbance [31]. However, in spite of all the milestones in AD research, it still has no cure, with high prevalence as well as huge economic burdens. Additionally, some detrimental effects have been associated with the AD-approved medications earlier stated. This necessitates the need for a better alternative in preventing or managing AD. Hence, this study assessed the therapeutic potentials of WIN on AlCl_3−_ and d-gal-induced rat models with cognitive impairments. The cognitive functions of the rats were evaluated through MWM, while oxidative stress and neurogenesis parameters were evaluated through ELISA. Finally, structural changes on the CA1 region of their hippocampus was evaluated through Nissl’s staining.

## 2. Materials and Methods

### 2.1. Animals

Healthy, male albino Wistar rats, two to three months old weighing 200–350 g were utilized in this study. The rats were obtained from a local supplier, Bistari Enterprise, Taman Sri Serdang, Seri Kembangan, Selangor, Malaysia. They were housed 2–3 rats per cage under the same laboratory conditions (temperature 22 ± 25 °C, 12 h light: 12 h dark cycle) for one (1) week Acclimatisation. Rats were fed with standard commercially available rats chow (Gold coin feed mills, Malaysia) and water ad libitum. The test protocols adopted for all the experiments were in line with the principles of laboratory animal care and approved by the Universiti Putra Malaysia Institutional Animal Care and Use Committee (IACUC Reference Number: UPM/IACUC/AUP-R097/2018).

### 2.2. Experimental Design

**Figure 1:**
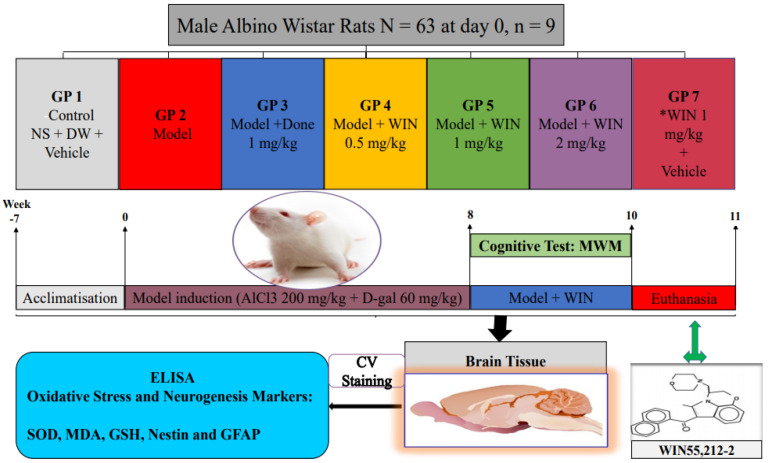
Experimental design to test rat’s cognitive function, check the level of damage on the hippocampus and quantify oxidative stress and neurogenesis markers following the induction of cognitive impairments by AlCl_3_ and d-gal co-administered with WIN. SOD—superoxide dismutase, MDA—malonaldehyde, GSH—glutathione, GFAP—glial fibrillary acidic protein, CV Staining—Cresyl Violet Acetate, GP—group, NS—normal saline, DW—distilled water, Model—AlCl_3_ + d-gal, AlCl_3_—Aluminium chloride, d-gal—d-galactose, Done—Donepezil, MWM—Morris Water Maze, WIN—synthetic cannabinoid agonist, WIN55,212-2.

The rats were randomly divided into seven groups, comprising of the control and 6 treatment groups (Figure 1). The dosage of donepezil 1 mg/kg administered was adapted from [32], while the dosage of WIN administered was selected based on the literature researched [33,34]. Donepezil is used for symptomatic treatment of AD, this drug is a specific inhibitor of an enzyme AChE, whose main physiological function is the hydrolysis of neurotransmitter acetyl choline (ACh) [35]. Thus, the donepezil administered group serves as a positive control group in this study in order to compare its effects to WIN. The protocol lasted for ten weeks, before the completion of the regimen.

### 2.3. Morris Water Maze Test

The Morris water maze test is a task primarily conceived to assess the spatial learning and memory paradigm of cognitive impairments in rodents [36,37,38]. MWM is a type of test extensively used and well-accepted for spatial learning in mice and rats, wherein the rodents depend on the localisation of distal visual signs to navigate from start points around the perimeter of an open swimming arena to find a submerged platform [39,40]. The latency, distance traversed by the rodents and the swimming speed to locate the submerged platform are recorded using video and are the general measure considered in this task [41]. Spatial learning is measured across a set of repeated trials whereas reference memory is evaluated by a preference for the platform area (the quadrant targeted) after the platform is taken off [38].

In the 9th week of continuous treatment, a day before Morris Water Maze (MWM) test began, each of the rats were placed in the tank used for the MWM test which was filled with water and let to swim for 120 s towards a wall for acclimatization and also to select out those rats that could not swim. MWM test is a well-known assay of hippocampal-dependent spatial memory, the place navigation and spatial probe tests were the two parts of the MWM test [38,40]. This paradigm assesses the rat’s ability to learn and recollect the spatial position of a submerged platform in a circular pool of water. The MWM tank’s dimension is as follows; diameter 122 cm, height 62.5 cm, filled with water devitrified by milk to a height of 40 cm and maintained at a constant temperature of 24 ± 2 °C [42,43]. A video tracking camera was placed above the MWM tank and coupled to the laptop with software (ANY-maze TM, Stoelting Co., Chicago, IL, USA). With the aid of the software, the tank was virtually separated into four equal quadrants. A platform measuring 10 cm in diameter was placed in the middle of one of the quadrants (now called the original quadrant). On the first day of the test, the platform (escape platform) was kept visible to the rats; 2 cm above the water level, and in the following five days the escape platform was hidden to the rats (submerged 2 cm below the water level) while the escape platform quadrant was retained for the five days of the test. The same procedure was followed for the reversal only that the platform was transferred to the opposite quadrant where it was kept during the first cycle (forward) of the MWM test. The test was carried out in line with the protocol proposed by Vorhees and Williams, 2006 with slight modification. On the first day of the test, the rats were trained to locate the visible escape platform and for the next five successive days, the rats were trained to trace the submerged escape platform. During the place navigation aspect of MWM, each rat undertook four training sessions daily. In each session, rats were randomly placed in any of the three quadrants (starting points) other than the one with the escape platform and allowed to find the submerged escape platform with a maximum time of 120 s given for each trial. This was repeated for the other two quadrants thus making the four trial sessions. Rats that were able to find the escape platform were allowed to stay on it for 20 s at the end of each trial so as to enable them to familiarise with the location of the escape platform very well. Whereas those rats that failed to locate the escape platform were guided gently to the platform and allowed to remain there for 20 s as well. The spatial probe test was conducted 24 h after the place navigation test ended. In this test the escape platform was taken away, rats were separately placed into the water tank at the quadrant opposite to the original quadrant and allowed to search for the removed escape platform for 30 s. At the end of the 30 s the rats were removed from water, dried with a towel and taken back to their home cage. Parameters such as escape latency, time spent in the target quadrant, the speed of navigation were all recorded by a video camera linked to a computer with ANY-maze software for analysis.

### 2.4. Nissl Staining and Scoring

Samples of hippocampi were isolated from the rats’ brains and stained with Nissl stain. Nissl’s staining was performed to count the number of viable neurons in the rats’ hippocampus, the whole brains of the rats were removed and were fixed for five days in 10% neutral buffered formalin. These fixed brain tissues were placed into tissue cassettes and dehydrated using an automated tissue processor. These processed brain tissues were then embedded in paraffin blocks using an embedding machine. Through the use of a microtome, coronal sections of the tissue blocks were sectioned (6 μm) forming a continuous ribbon of tissue sections. These ribbons were gently collected and placed in water in a water bath (38 °C) to straighten out the sections prior to fishing them on superb-frosted glass slides. The tissues were then de-paraffinised in an oven for 60 min at 70 °C and then in xylene for 5 min and rehydrated in graded alcohol (95%, 70%) for 3 min for each concentration and lastly in distilled water for 3 min. The tissue sections were stained with 0.1% cresyl violet acetate solution at 60 °C in an oven for 10 min to assess the extent of neuronal damage in the rats’ brains. The slides were later washed in distilled water for 3 min and dehydrated in graded alcohol (70%, 95%, and 100%) for 3 min in each concentration of alcohol. After dehydration, the tissues were kept in xylene for 5 min prior to being mounted with dibutyl phthalate xylene (DPX), a mounting media and covered with a cover slip.

Image acquisitions were made using a high-resolution compound microscope, pathologist grade Olympus BX51TRF-CCD and with the help of Dino eye (Dino lite, Dunwell Tech Inc., Torrance, Los Angeles, CA, USA). All the slides were examined and a systematic scoring method was performed at 400× *g* magnifications respectively and 4 to 6 spots were randomly selected. On each spot, hippocampal neuronal cells with intact spherical and finely stained nuclei were counted as viable ones [44].

For the purpose of scoring, with a guide of an independent pathologist, at least five sections of the hippocampus were taken for evaluation in a minimum of three rats per group (*n* = 3). Additionally, the number of viable cells were averaged in at least five fields under view in the mid portions of the Cornus ammonus 1 (CA1) sub-field of the hippocampus. The presence of Nissl-stained dark neurons were evident, whereas the number of viable cells in the CA1 sub-field of the hippocampus were counted. The proportion of neuronal loss was evaluated as the mean number of neurons in the control sections minus the mean number of neurons in treatment group sections divided by the mean number of neurons in control sections times 100 [45,46,47]. Scoring was conducted as indicated in Figure 5 in the results section.

### 2.5. Preparation of Brain Tissue Samples

The rats were euthanised by decapitation and brains were taken out and washed with ice-cold saline. The brain samples used for histology were preserved in 10% neutral buffered formalin for subsequent use. For biochemical analysis, the brains of the rats were isolated and then homogenised with ice-cold radioimmunoprecipitatation assay (RIPA) buffer (200 µL) supplemented with protease inhibitor (20 µL) in a volume of 10 times the weight of the tissue to prepare 10% hippocampal and cerebral homogenates. Using a refrigerated centrifuge (Zentrifugen, Hettich, Westphalia, Germany), the homogenates were centrifuged at 12,000× g for 10 min at 4 °C and the supernatant was aliquoted and kept at −80 °C and subsequently used for biochemical analysis.

### 2.6. Measurement of Protein

Total protein concentration of the rat brain tissues was calculated using the bicinchoninic acid assay (BCA assay). Bovine serum albumin (BSA) (1 mg/mL) was taken as a standard in the range of 0.01–0.1 mg/mL for comparison with the total protein.

### 2.7. Measurements of Biochemical and Neurogenesis Parameters

The quantitative measurement of MDA, SOD, GSH GFAP and Nestin levels in the hippocampi and the prefrontal cortex tissues of rats were evaluated through ELISA and colorimetric assays according to the user’s manual (Elabscience, Houston, TX, USA and FineTest, Wuhan, Hubei, China).

## 3. Results

### 3.1. WIN Attenuated AlCl_3−_ and d-gal-Induced Cognitive Impairments in Rats during MWM Test

**Figure 2:**
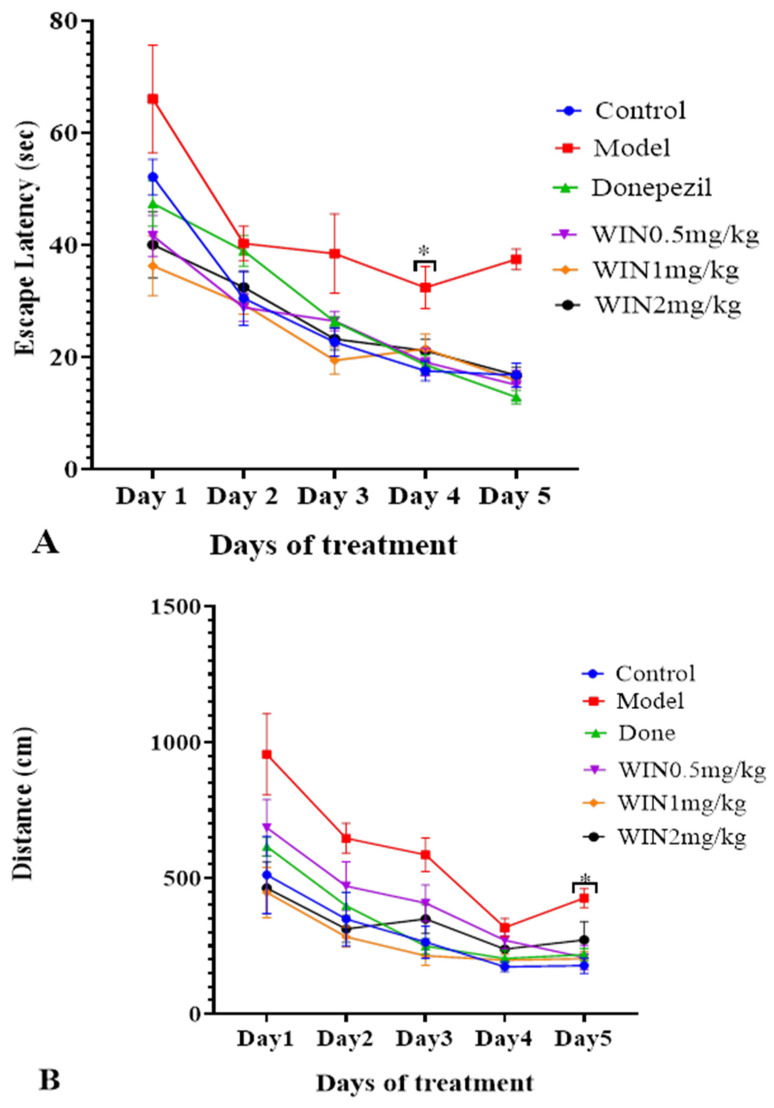

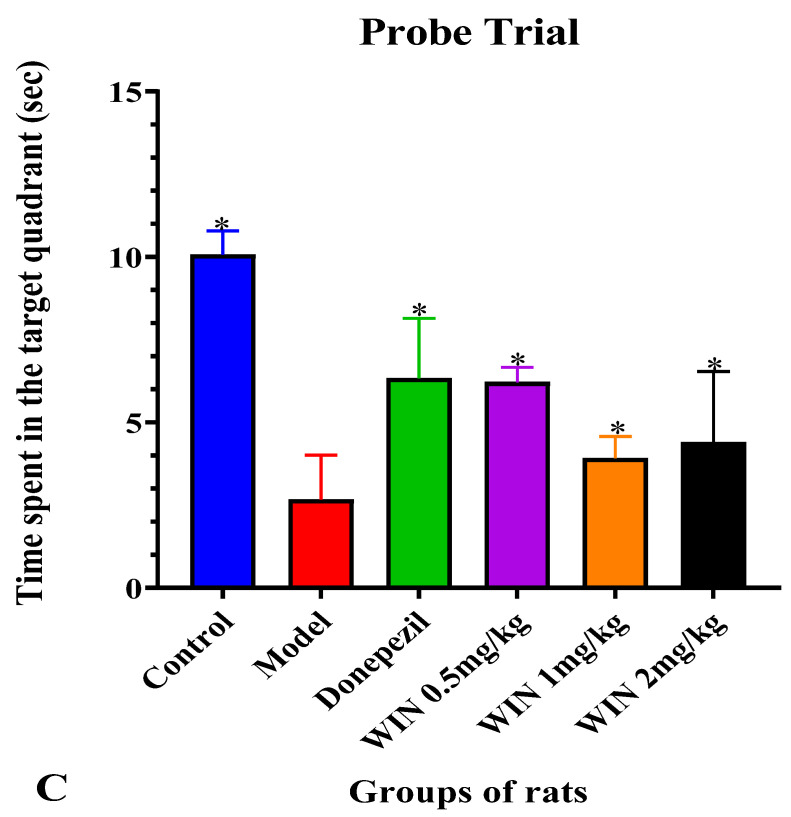
Assessment of spatial learning and memory of rats in Morris water maze. (**A**) Escape latency (s) during the 5 days trial to locate the hidden platform as part of the learning phase in the MWM. (**B**) The average distance traversed by the groups of rats over the 5 days trial. (**C**). The probe trial was conducted on day 6 after the trial phase when the hidden platform was removed. Rats were allowed to search for the platform in the target quadrant. The experimental groups include; Normal saline + Distilled water, (control), Model (AlCl_3_ 200 mg/kg + d-gal 60 mg/kg), positive control (Model + Donepezil 1 mg/kg, positive control), WIN low (Model + WIN55,212-2 0.5 mg/kg), WIN medium (Model + WIN55,212-2 1 mg/kg) and WIN high (Model + WIN55,212-2 2 mg/kg). Data were expressed as ± SEM for (*n* = 6) rats per group. Statistical analysis was performed by two-way repeated measures ANOVA in (**A**,**B**), and One-way ANOVA in (**C**). * *p* < 0.05 vs. model group.

**Figure 3:**
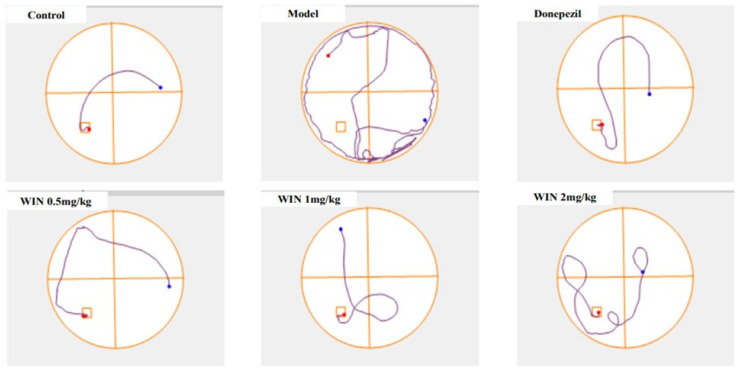
Representative images of track plots of spatial learning in AlCl_3_ and d-gal-induced cognitive impaired rats on day 5. The experimental groups include; group Normal saline + Distilled water, (control), Model (AlCl_3_ 200 mg/kg + d-gal 60 mg/kg), positive control (Model + Donepezil 1 mg/kg, positive control), WIN low (Model + WIN55,212-2 0.5 mg/kg), WIN medium (Model + WIN55,212-2 1 mg/kg) and WIN high (Model + WIN55,212-2 2 mg/kg), *n* = 6 for each group.

The MWM test was conducted to assess the therapeutic effect of cannabinoid receptor, WIN on AlCl_3_ and d-gal-induced cognitive impairments in rats. Figure 2A,B are timeline graphs showing 5 days average escape latency and distance covered by the various groups of rats to locate the submerged escape platform. All the rat groups exhibited decreases in time spent and distance covered to locate the submerged platform as the days progresses, indicative of learning and memory by the rats. Nevertheless, the learning pattern changed on days 4 and 5 as the model group of rats spent a long time and covered a longer distance before they located the submerged escape platform. Two-way ANOVA indicated statistically significant differences in the interactions between the effects of treatment and days of treatment, [F (5, 30) = 11.43, *p* = 0.001] for the escape latency of the rats (Figure 2A). Tukey’s post hoc comparison revealed statistically significant different decreases (*p* < 0.05) in time to find the submerged escape platform on day 4 and day 5 by the control, donepezil WIN55,212-2 0.5 mg/kg, WIN55,212-2 1 mg/kg and WIN55,212-2 2 mg/kg rats’ groups in comparison with the model group. Additionally, two-way ANOVA showed statistically significant differences in the interactions between the effects of treatments and the days of treatment, [F (5, 30) = 7.332 *p* = 0.001] for the average distances covered by the various rat groups (Figure 2B). Tukey’s post hoc indicated statistically significant different increases (*p* < 0.05) in the distance covered by the model group of rats on days 1–3 when compared to the rats in the control, donepezil, WIN55,212-2 0.5 mg/kg, WIN55,212-2 1 mg/kg and WIN55,212-2 2 mg/kg groups. Parallel trend was also observed on day 5 of the test where statistically significant different increases were observed in the distances covered by the model group of rats in comparison with the control, donepezil and WIN55,212-2 (0.5 mg/kg, 1 mg/kg and 2 mg/kg) groups. Figure 2C shows the time spent by the various groups of rats in the target quadrant looking for the hidden platform taken off on day 6 of the MWM test. The rats in the model group spent relatively less time in the target quadrant searching for the removed escape platform. One-way ANOVA [F (5, 30) = 3.746, *p* = 0.009] exhibited statistically significant differences in the times spent in the target quadrant among the various rat groups. Tukey’s post hoc comparison indicated statistically significant different increases in the times spent in the target quadrant by the control (10.07 ± 0.7191, *p* = 0.009), donepezil (6.333 ± 1.815, *p* = 0.009), WIN55,212-2 0.5 mg/kg (6.217 ± 0.4445, *p* = 0.009), WIN55,212-2 1 mg/kg (3.917 ± 0.6585, *p* = 0.009) and WIN55,212-2 2 mg/kg (4.400 ± 2.138, *p* = 0.009) rats’ groups in comparison with the model group (2.667 ± 1.341, *p* = 0.009). The track plot for the MWM test is shown in Figure 3, the pathway followed by the model group of rats before reaching the escape platform is longer when compared to the control group of rats. The rats in the model group were going around the pool of water by the edges, while WIN- and donepezil-treated groups of rats were roaming purposefully in the centre of the pool searching for the hidden platform.

### 3.2. Histological Findings

**Figure 4:**
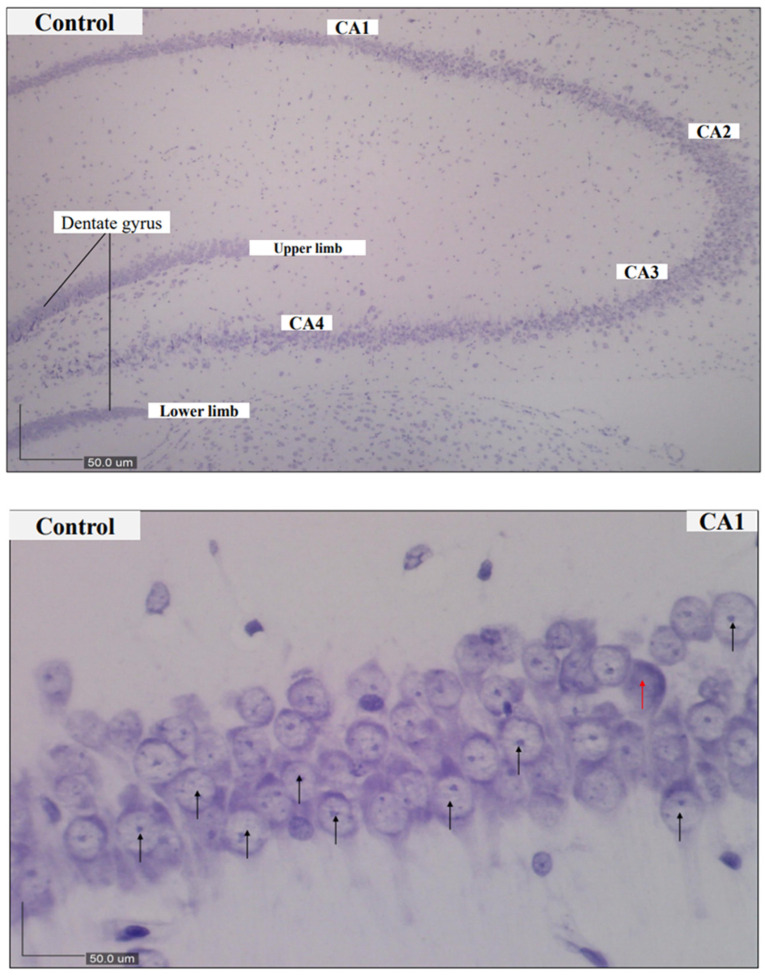

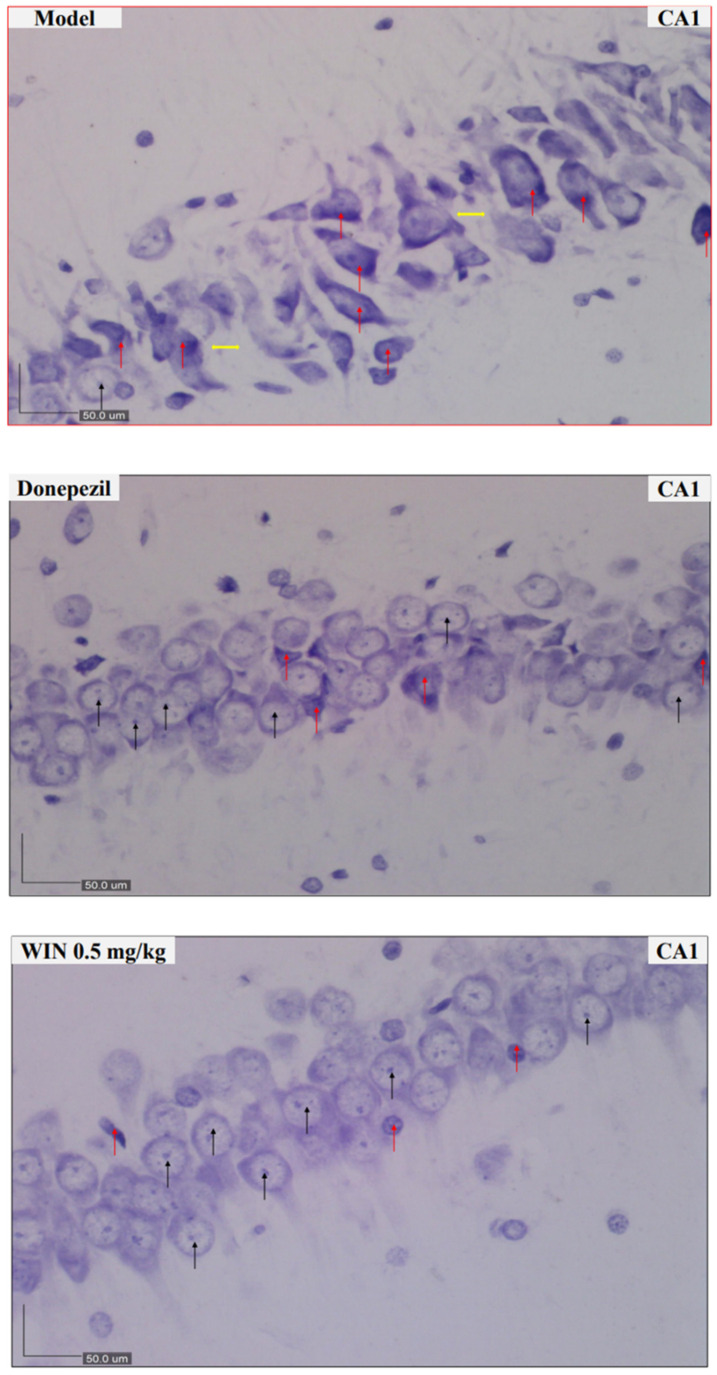

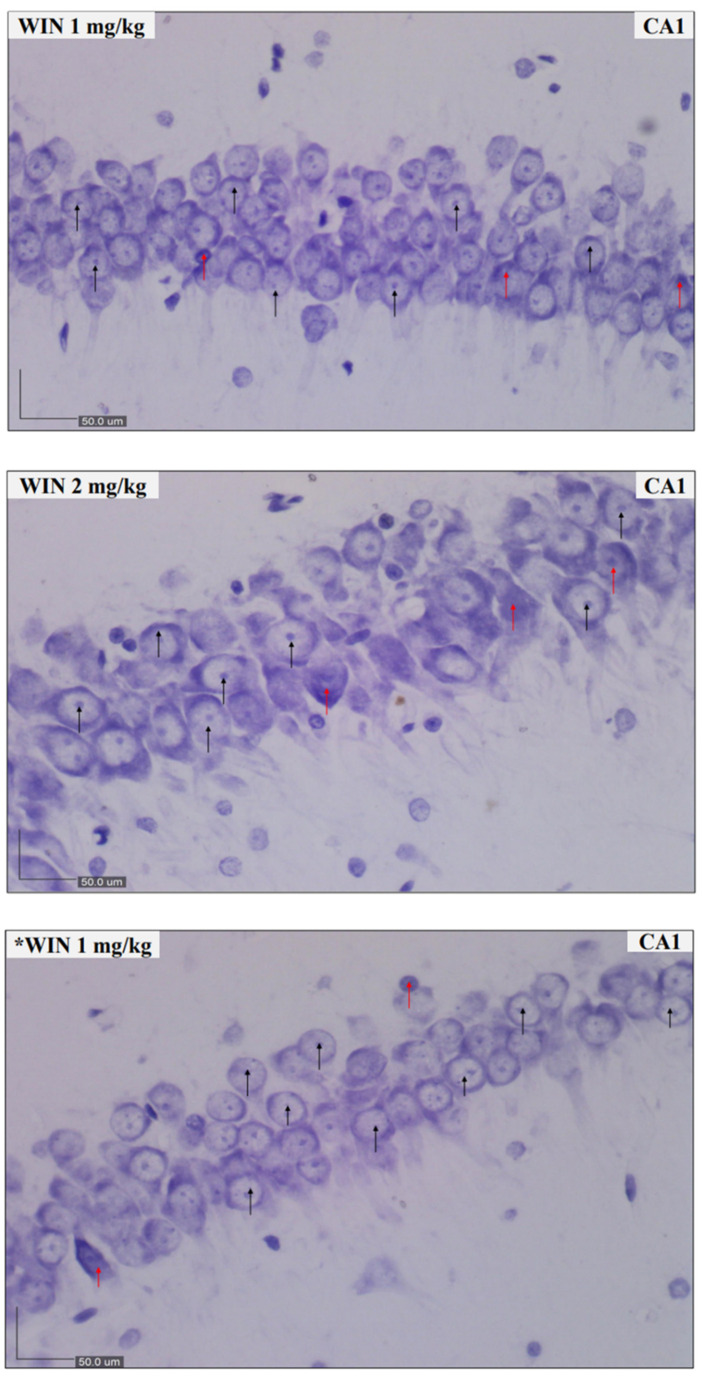
Representative photomicrographs of brain sections of rats (CA1 subfield of the hippocampus) stained by cresyl violet. Control, CA1–CA4—Rat hippocampus revealing all regions, (magnification ×100). Control, CA1—Control group rats showing normal histology of pyramidal cells arranged in a dense layer. Model, CA1—Model group rats indicating degenerated pyramidal cells and distortion of pyramidal layer. Donepezil, CA1—Donepezil group showing thinning of pyramidal layer and a few degenerated cells. WIN 0.5 mg/kg, WIN 1 mg/kg and *WIN 1 mg/kg (CA1) rats’ group respectively showing abundant of normal pyramidal cells similar to the control group. Whereas the rats treated with WIN 2 mg/kg, CA1 display few pyramidal cells suggestive of alterations in the arrangement of the pyramidal cells in this group of rats. Magnification for Control, CA1-CA4 is ×100, while Control, CA1, Model, CA1, Donepezil, CA1, WIN 0.5 mg/kg, CA1, WIN 1 mg/kg, CA1, WIN 2 mg/kg, CA1 and *WIN 1 mg/kg, CA1 is ×400, black arrows pointing at viable neurons and red arrows pointing at dead neurons whereas yellow arrows indicate spaces (altered neuronal arrangement). CA1 = cornu ammonis 1; CA2 = cornu ammonis 2; CA3 = cornu ammonis 3; CA4 = cornu ammonis 4; DG = Dentate gyrus.

Morphological analysis (Nissl’s staining and light microscopy) of their brains were carried out and marked neuronal loss was evident in the model whereas the histomorphological profile of the control was intact in Figure 4. Micrographs of the control groups indicated a condensed layer of pyramidal cells with vesicular nuclei, signifying viable neurons. Obvious alterations of the pyramidal cell layer and degenerations of the cells as revealed by hyper chromic neurons were seen in the CA1 sub-field of the model group of rats whereas a dense layer of pyramidal cells associated with vesicular nuclei indicating the presence of viable cells was observed in the treatment groups which reflected was is obtainable in the control groups of rats.

**Figure 5:**
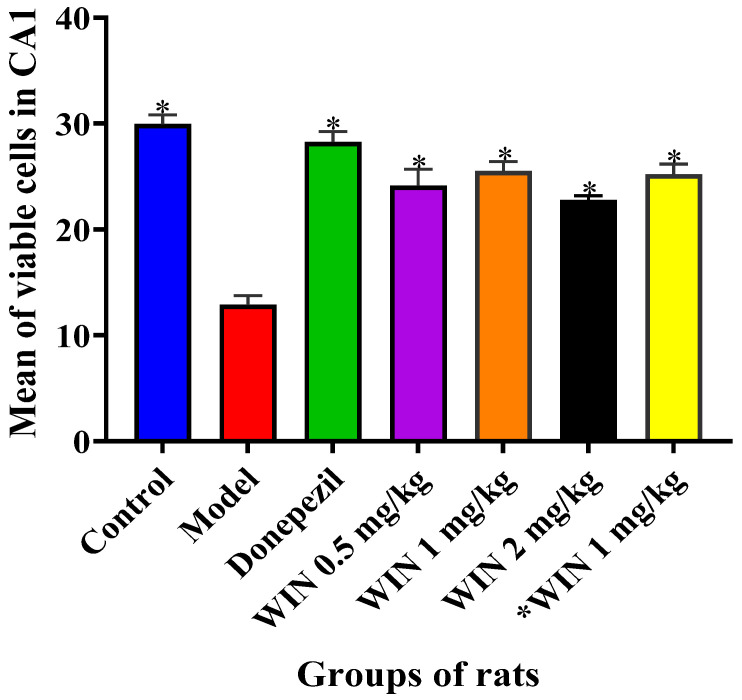
Significant differences in the number of viable cells counted in all the sub-fields of the hippocampus. The experimental groups include, control = normal saline + distilled water, Model (AlCl_3_ 200 mg/kg + d-gal 60 mg/kg), Donepezil (model + d-gal 60 mg/kg + Donepezil 1 mg/kg), WIN 0.5 mg/kg (model + WIN55,212-2 0.5 mg/kg), WIN 1 mg/kg (model + WIN55,212-2 1 mg/kg), WIN 2 mg/kg) (model + WIN55,212-2 2 mg/kg) and *WIN 1 mg/kg (WIN55,212-2 1 mg/kg). One-way ANOVA followed by Tukey’s post hoc test among the groups of rats. * *p* < 0.05 vs. control. Data were expressed as SEM ± *n* = 4.

One-way ANOVA (Figure 5) showed statistically significant differences between the various groups of rats [F (6, 21) = 29.99, *p* = 0.001] in the number of viable cells in CA1. The Tukey’s post hoc test (*p* < 0.05) revealed a statistically significant decrease in the number of viable cells within the CA1 subfields the model group of rats, and the six other treatment groups: Model (12.88 ± 0.8809, *p* = 0.001), Donepezil (28.25 ± 1.005, *p* = 0.892), WIN 0.5 mg/kg (24.13 ± 1.573, *p* = 0.008), WIN 1 mg/kg (25.50 ± 0.9186, *p* = 0.066), WIN 2 mg/kg (22.75 ± 0.4564, *p* = 0.001) and *WIN 1 mg/kg (25.19 ± 0.9915, *p* = 0.042) when compared with the control group control (29.94 ± 0.8919, *p* = 0.001).

### 3.3. Biochemical Findings

#### 3.3.1. Effects of WIN on MDA Levels in the Brains of AlCl_3_ and d-gal-Induced Rats

**Figure 6:**
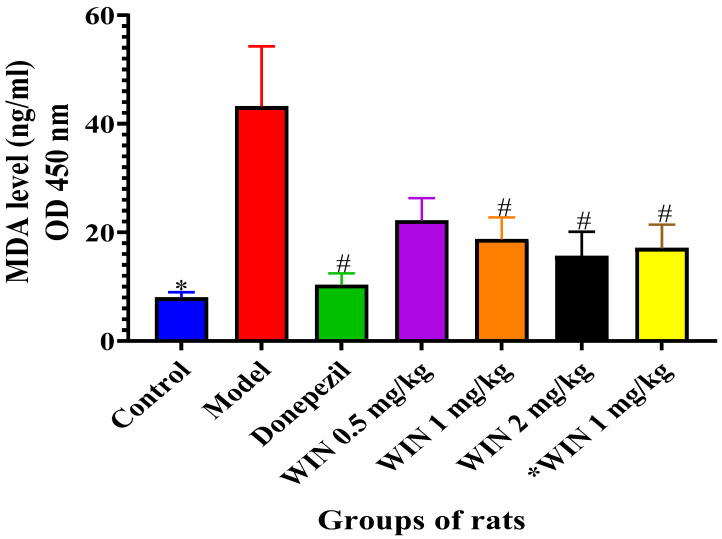
Effects of WIN on MDA activities in the brains of d-gal- and AlCl_3_-induced rats. Values are shown as mean ± SEM, *n* = 5. * *p* < 0.05 vs. model, # *p* < vs. control.

Higher levels of MDA are one of the known pointers of oxidative damage in the brains of rats, and MDA levels were quantified in the rat brains as indicated in Figure 6. The model rat groups displayed a significant increase in the MDA levels in the brain when compared to the control, donepezil and the WIN co-administered groups of rats. One-way ANOVA showed statistically significant differences in MDA levels in the brain of the rats [F (6, 28) = 4.733, *p* = 0.002]. Tukey’s post hoc showed statistically significant different increases of MDA levels in the brain of the model group of rats (72.69 ± 11.03, *p* = 0.001), in contrast to the control group (10.51 ± 0.9980). Treatment of rats with donepezil 1 mg/kg/day reduced MDA levels (17.34 ± 2.134, *p* = 0.003) in their brains. Additionally, MDA levels in d-gal and ACl_3_-induced rats were decreased by the treatment with WIN 1 mg/kg (31.35 ± 4.048, *p* = 0.042), WIN 2 mg/kg (30.92 ± 4.468, *p* = 0.016) and *WIN 1 mg/kg (32.68 ± 4.303, *p* = 0.026) in comparison with the model group of rats (72.69 ± 11.03, *p* = 0.001) (Figure 6). No statistically significant differences were observed between the control, donepezil, WIN 0.5 mg/kg, WIN 1 mg/kg, WIN 2 mg/kg and *WIN 1 mg/kg treated group of rats. Tukey’s post hoc showed statistically significant different increases in MDA levels (72.69 ± 11.03, *p* = 0.001) in the cerebral cortex of the model group of rats when compared to the control group (10.51 ± 0.9980, *p* = 0.001). Finally, decreases in MDA levels were also seen in cortex of the donepezil treated group of rats (17.34 ± 2.134, *p* = 0.003), in comparison with the model group of rats (72.69 ± 11.03, *p* = 0.001).

#### 3.3.2. Effects of WIN on SOD Activities in the Brain of AlCl_3_ and d-gal and Induced Rats

**Figure 7:**
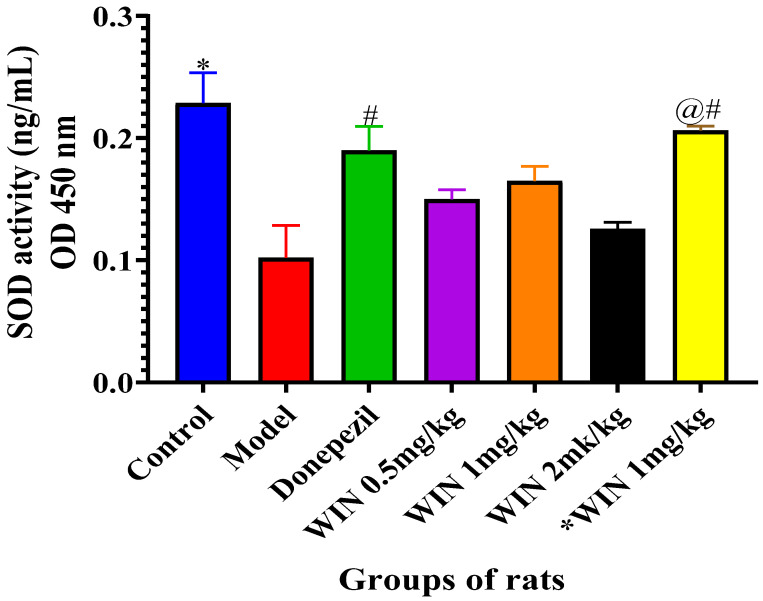
Effects of WIN on SOD activities in the brains of AlCl_3−_ and d-gal-induced rats. Values are shown as mean ± SEM, *n* = 5. * *p* < 0.05 vs. model, # *p* < vs. control and @# *p* < vs. model.

To evaluate the antioxidant effects of WIN in d-gal- and AlCl_3_-induced rats, the activities of SOD in the rat’s brains were quantified. Figure 7 shows the SOD activities in both the hippocampus and the cerebral cortex of the various groups of rats, with a marked decrease in the model groups of rats. However, co-administration with donepezil and different doses of WIN reversed the increase. One-way ANOVA indicated statistically significant differences of SOD activities in the cerebral cortex of the various groups of [F (6, 28) = 7.156, *p* < 0.001]. Likewise, Tukey’s post hoc showed statistically significant differences in the reduction of SOD activities in the model group (0.1440 ± 0.02648, *p* = 0.001) in comparison with the control group of rats (0.2605 ± 0.02509, *p* = 0.001). Whereas, increases in SOD activities in the control (0.2605 ± 0.02509, *p* = 0.001) and *WIN 1 mg/kg (0.2112 ± 0.003609, *p* = 0.002), groups of rats were observed, in contrast to the model group (0.1440 ± 0.02648, *p* = 0.001) (Figure 7). Similarly, Tukey’s post hoc also showed statistically significant differences in the decrease in SOD activities in the model group (0.1440 ± 0.02648) when compared to the control group of rats (0.2606 ± 0.02509). Furthermore, there was statistically significant differences in the SOD activities observed between the control (0.2606 ± 0.04169, *p* = 0.001) group of rats, when compared to WIN 0.5 mg/kg (0.1577 ± 0.007738, *p* = 0.036), WIN 2 mg/kg (0.1378 ± 0.005488, *p* = 0.003) (Figure 6). However, there were no statistically significant differences in the SOD activities observed between the control (0.2606 ± 0.04169, *p* = 0.001) group of rats, when compared to the donepezil (0.2377 ± 0.01976, *p* = 0.663) group of rats.

#### 3.3.3. WIN Potentially Enhances Reduced Glutathione (GSH) Concentrations in the Brain of AlCl_3_ and d-gal-Induced Rats

**Figure 8:**
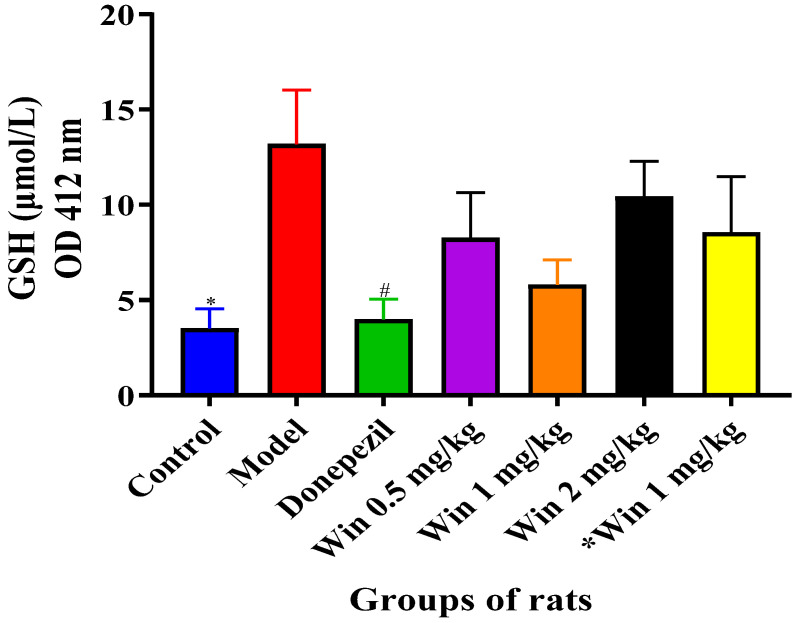
WIN potentially reduced GSH level in the cerebral cortex of d-gal- and AlCl_3-_induced rats. Values are presented as mean ± SEM, *n* = 5. * *p* < 0.05 vs. model, # *p* < vs. control.

To measure the effects of WIN in d-gal- and AlCl_3_-induced rats, the level of reduced GSH in the rat’s brains was evaluated. Figure 8 shows a reduced GSH level, with a marked increase in the model group of rats. However, co-administration with donepezil and different doses of WIN reversed the increase. One-way ANOVA showed statistically significant differences in GSH level in the cerebral cortex of the various groups of rats [F (6, 35) = 2.799, *p* = 0.025]. Similarly, Tukey’s post hoc revealed statistically significant differences in the increase in GSH level in the model group (22.00 ± 2.837, *p* = 0.025) in contrast to the control group of rats (5.867 ± 1.028, *p* = 0.025). Additionally, there were no statistically significant differences in the GSH level observed between the control (5.867 ± 1.028, *p* = 0.025) group of rats, in comparison with the donepezil (6.615 ± 1.061, *p* = 0.048) group of rats.

#### 3.3.4. WIN Enhances Nestin Level, a Marker for Neurogenesis in the Brain of AlCl_3−_ and d-gal-Induced Rats

**Figure 9:**
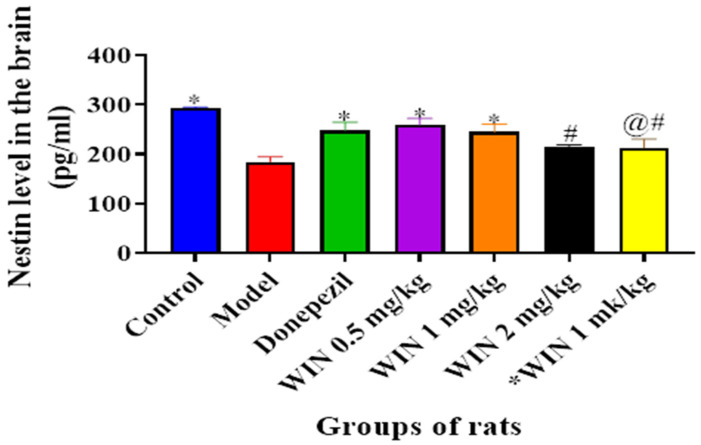
Effects of WIN on Nestin levels in the brain of d-gal- and AlCl_3_-induced rats. Value are shown as mean ± SEM, *n* = 5. * *p* < 0.05 vs. model group, # *p* < 0.05 vs. control and @# *p* < 0.05 vs. donepezil group.

Figure 9 shows the ELISA results for Nestin levels in the brain of the various rats’ groups, in which a marked decrease in Nestin levels was observed in the model group of rats. Statistically significant differences in Nestin levels were observed among the various rat groups [F (6, 14) = 7.522, *p* = 0.001] as shown by one-way ANOVA. Tukey’s post hoc indicated statistically significant differences in the increase in Nestin level in the control group (297.5 ± 2.525, *p* = 0.001), donepezil (265.2 ± 16.96, *p* = 0.048), and WIN 0.5 mg/kg/day (273.9 ± 13.41, *p* = 0.016) groups of rats in contrast to the model group (203.8 ± 10.07, *p* = 0.001) (Figure 9). However, Tukey’s comparison did not indicate statistically significant decreases of Nestin levels in WIN 1 mg/kg (274.0 ± 15.59, *p* = 0.063), WIN 2 mg/kg (219.2 ± 4.894, *p* = 0.678) and *WIN 1 mg/kg/day (248.5 ± 18.56, *p* = 0.732) groups of rats.

#### 3.3.5. WIN Enhances the Level of Neurogenesis Marker Glial Fibrillary Acidic Protein (GFAP) in the Brain of AlCl_3−_ and d-gal-Induced Rats

**Figure 10:**
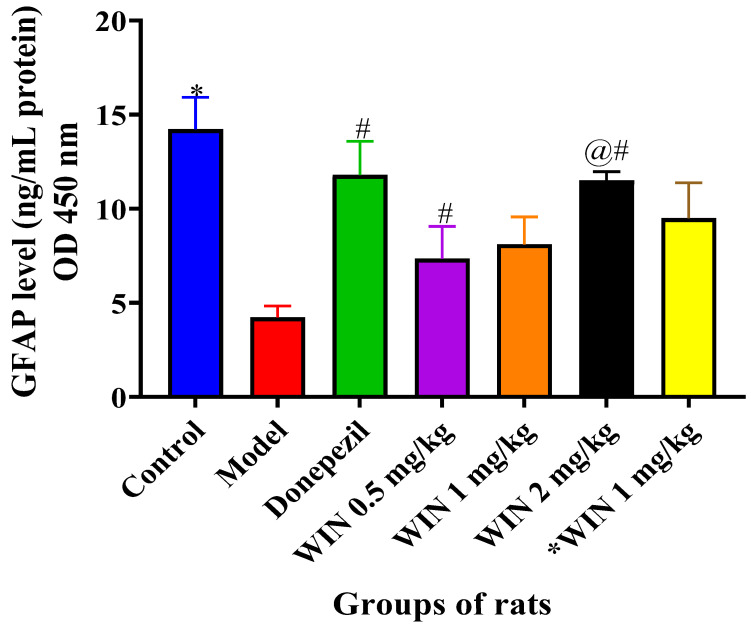
Effects of WIN on GFAP levels in the brain of d-gal- and AlCl_3_-induced rats. Value are reported as mean ± SEM, *n* = 5. * *p* < 0.05 vs. control group, # *p* < 0.05 vs. donepezil and @# *p* < 0.05 vs. WIN2 mg/kg group.

Figure 10 shows the ELISA results for GFAP levels in the brain of the various rats’ groups, in which a marked decrease in GFAP levels were observed in the model group of rats. Statistically significant differences in GFAP levels were observed among the various rat groups [F (6, 35) = 5.032, *p* = 0.001] as shown by one-way ANOVA. Tukey’s post hoc indicated statistically significant differences in the increase in GFAP level in the control group (18.35 ± 1.698, *p* = 0.001), donepezil (16.24 ± 1.795, *p* = 0.015) and WIN 2 mg/kg/day (12.51 ± 0.4661, *p* = 0.021) groups of rats in contrast to the model group (6.098 ± 0.6128, *p* = 0.001) (Figure 10). However, Tukey’s comparison did not indicate statistically significant decreases of GFAP levels in WIN 0.5 mg/kg (12.04 ± 1.717, *p* = 0.746), WIN 1 mg/kg (12.47 ± 1.456, *p* = 0.518) and *WIN 1 mg/kg/day (15.44 ± 1.881, *p* = 0.182) groups of rats.

## 4. Discussion

Earlier studies have revealed that chronic administration of d-gal- or AlCl_3_-induced changes that resembled natural ageing processes in rats, such as dysfunction in the cholinergic system [48], cognitive impairment [49,50,51], oxidative stress [52,53] and advanced glycation end products formation [23] with biochemical and pathological changes of astrocytes [54]. It was found that combined administration of AlCl_3_ and d-gal in rats induced cognitive dysfunction and degeneration of pyramidal cells in their hippocampus [43,55]. In this study, rats induced with AlCl_3_ and d-gal exhibited obvious impairments in their learning and memory, and there were histomorphological alterations of their hippocampal neurons. Thus, the outcomes of the current study demonstrate that this rat model could be a good candidate for the study of some AD-related pathologies. Furthermore, cresyl violet staining indicated that neurons in rats in the control group were arranged in a parked manner as evident by clear nucleoli (Figure 4 CA1 WIN 1 mg/kg and WIN 2 mg/kg respectively). In contrast, hippocampal CA1 neurons of rats treated with AlCl_3_ and d-gal were morphologically perturbed and had significantly increased distortions of the pyramidal cells as indicated by darkly stained cells in Figure 4 CA1, Model. The number of neurons with cytoplasmic shrinkage and pyknotic nucleus was also increased. Following co-administration with WIN, the number of pyknotic neurons significantly reduced, and the extent of structural aberrations was lower than that of the AlCl_3_ and d-gal groups. This is consistent with the recent findings of Wang and others where WIN improved bilaterally common carotid arteries occlusion in rats [56]. Administration of WIN in this work signified a vital role in ameliorating dementia and morphological alterations caused by AlCl_3_ and d-gal in the rats. Based on the available information at our disposal, the results of the current work are the first to report on the histomorphological regenerative abilities and powers in rats treated with WIN following cytoarchitectural aberrations caused by AlCl_3_ and d-gal.

Structurally, the chronic exposure to d-gal and AlCl_3_ in the current study resulted in apparent structural changes, such as higher numbers of pyknotic cells and disorientation of the pyramidal cell layer arrangements, in the hippocampus of rats. Whereas the treatment with WIN amended the changes observed. Therefore, it is informative to report that results of the present study unraveled that WIN had a cytoprotective potential in preserving normal cytoarchitecture, perhaps suggesting that it could bring about neurogenesis in aged rats’ neurons [33]. Hippocampal cells were commonly used in previous studies on neuroapoptosis, which were frequently measured by the expression of cleaved caspase-9 and cleaved caspase-3 [57,58]. In this work, the morphological indications of neuroapoptosis were evidently indicated by directly observing the cytoarchitectural variations in the cells of the hippocampus of rats using a high-resolution microscope and with the aid of Dino eye (Dino-lite software). Dino eye is a piece of equipment that enhances the magnifications as well as the clarity of the tissue under view. The relative potencies of the WIN in ameliorating the histomorphological aberrations in the present study are in consensus with the earlier report of [33] as well as a recent finding by [59].

The oxidative stress-mediated neurotoxicity is a major pathological feature in the underlying neurogenerative mechanism of AD [60]. Furthermore, oxidative stress is largely considered an early event that cascades the occurrence of neurodegenerative disorder and plays a crucial part in Aβ-induced cell death in the brain [61]. Oxidant-level-related dysfunctions have been reported in AD patients, which could be attributed to either hyperproduction of oxidants or deficit in antioxidants. Higher levels of MDA are one of the known pointers of oxidative damage in the brains of rats, and MDA levels were quantified in the rat brains as indicated in Figure 6. Moreover, mounting evidence from preclinical and clinical studies suggests that oxidative stress is linked with etiopathology of AD [62,63], which includes mitochondrial dysfunction [64], increased Aβ-mediated neurotoxicity [65], and synaptic dysfunction and neuronal apoptosis [66]. Oxidants that were identified to be involved in the redox state include nitric oxide (NO), hydrogen peroxide (H_2_O_2_), hydroxyl radical (OH), hydroxyl anion (OH–) and peroxynitrite (ONOO–) [67]. In addition, a nexus between lipid peroxides levels, presence of senile plaques, antioxidants enzymes and accumulation of neurofibrillary tangles in brains of AD patients was reported [68]. Due to the strong connection between oxidative stress and cognitive dysfunction, chemical agents modulating ROS are thought to be critical in ameliorating cognitive deficits. In the current study, increased levels of MDA in the cerebral cortex and hippocampus were seen in AlCl_3−_ and d-gal-induced rats. However, administration of WIN had reduced the levels of MDA and increased SOD activities in the rat brains, suggestive of antioxidant properties of WIN. Furthermore, from the results analysed, there is an indication that WIN55,212-2 have exhibited therapeutic effect against oxidative stress and inhibit Aβ42 level thereby counteracting neuronal death. This could perhaps be due to beneficial effects of the WIN which may have antioxidant capacity and as a result of their interaction with various signaling pathways which regulate neuronal survival, differentiation and death. This is in agreement with the recent findings of [12,50,51,69,70] following AlCl_3−_ and d-gal-induced neurotoxicity in rats.

A decrease in neurogenesis in the hippocampus is an indication of age-related memory impairment in rats and humans [33]. In the current study, rats treated with AlCl_3_ and d-gal and showed reduced cellular activities in the brain, which was paralleled by obvious low GFAP levels in the brain. However, the administration of WIN or donepezil attenuated the aforementioned changes. Similarly, a growing body of knowledge points to Nestin as a unique intermediate filament that supports self-renewal ability in numerous subcategories of stem cells and progenitors, predominantly those of neural and mesenchymal origins. Nestin appears to have an effect on stem cell migration and differentiation, however, the knowledge on the underlying mechanisms is small [71]. To our knowledge, this is the first study that investigated the expression level of Nestin in this particular model of AD-like cognitive impairments following exposure to AlCl_3_ + d-gal treated with WIN55,212-2. In pre-clinical settings, previous work seems to have focused on immunofluorescence expressions of Nestin with few studies on its involvement in neurodegenerative diseases. Thus, little is also known on the involvement of Nestin in neurogenesis; its regulatory signaling pathways that control the expression and function of Nestin suggesting for future work in this regard. In addition to the current model already established, future improved in vivo models and detections tools to unravel the therapeutic applications of Nestin in different neurodegenerative diseases are of utmost importance.

## 5. Conclusions

Conclusively, administration of WIN55,212-2 ameliorated cognitive impairments in AlCl_3−_ and d-gal-induced rats’ model of AD as the administration of WIN55,212-2 reversed oxidative stress by reducing the levels of MDA and increasing the levels of SOD and GSH in the rats’ brains. Additionally, it elevates the levels of neurogenesis biomarkers, GFAP and Nestin, which is evident by a high number of viable neurons in the hippocampus of the WIN55,212-2-treated groups of rats. Therefore, this is a pioneer study that showed that synthetic cannabinoid agonist, WIN55,212-2 has the potential to partly restore neuronal loss in rats treated with AlCl_3_ and d-gal. However, further studies are required to understand the therapeutic potential of WIN55,212-2 in different experimental systems. Thus, protein analysis in order to uncover other mechanisms underlying the therapeutic effects of WIN in the AlCl_3−_ and d-gal-induced AD-like rat models in restoring neurogenesis need to be explored.

## Data Availability

Data will be available by request to the corresponding authors.

## Abbreviations

ACh	acetyl choline
AD	Alzheimer’s disease
AGES	Advanced glycation end products
AlCl_3_	Aluminium chloride
Aβ42	Amyloid beta 42
BCA	Bicinchoninic acid assay
BSA	Bovine serum albumin
ANOVA	Analysis of variance
CA1–4	Connus ammonis 1–4
ChEIs	Cholinesterase inhibitors
CV	Cresyl Violet Acetate
DG	Dentate gyrus
d-gal	d-galactose
DONE	Donepezil
DW	Distilled water
EC	Endocannabinoid system
GFAP	Glial Fibrillary Acid Protein
GP	Group
GSH	Glutathione
HRP	Horseradish peroxidase
H_2_O_2_	Hydrogen peroxide
IACUC	Institutional Animal Care and Use Committee
MDA	Malonaldehyde
MWM	Morris Water Maze
*n*	Number of rats per group
NMDA	*N*-methyl-d-aspartate
NO	Nitric oxide
NS	Normal saline
OD	Optical density
OH	Hydroxyl radical
ONOO–	Peroxynitrite
RIPA	Radioimmunoprecipitatation assay
ROS	Reactive oxygen species
SEM	Standard error of mean
SOD	Superoxide dismutase
USFDA	United States Food and Drugs Administration
T-GSH	Total glutathione
THC	Tetrahydrocannabinol
WIN	WIN55,212-2
